# Risk factors for subclinical atherosclerosis in HIV-infected patients under and over 40 years: a case–control study

**DOI:** 10.1186/1471-2334-13-274

**Published:** 2013-06-18

**Authors:** Valéria Maria Gonçalves Albuquerque, Josefina Claudia Zírpoli, Demócrito de Barros Miranda-Filho, Maria de Fátima Pessoa Militão Albuquerque, Ulisses Ramos Montarroyos, Ricardo Arraes de Alencar Ximenes, Heloísa Ramos Lacerda

**Affiliations:** 1 Department of Tropical Medicine, Universidade Federal de Pernambuco, Recife, Brazil; 2 Department of Clinical Medicine, Universidade de Pernambuco, Recife, Brazil; 3 Postgraduate in Medical Science, Universidade de Pernambuco, Recife, Brazil; 4 NESC Department, Centro de Pesquisas Aggeu Magalhães/FIOCRUZ, Recife, Brazil

**Keywords:** HIV, Risk factors, Atherosclerosis

## Abstract

**Background:**

Cardiovascular diseases (CVD) are a major cause of death in people with AIDS. Factors contributing to atherosclerosis include traditional risk factors, antiretrovirals and inflammatory factors related to HIV infection. This study set out to compare risk factors associated with subclinical atherosclerosis in individuals under and over 40 years of age.

**Methods:**

Case–control study with 697 HIV/AIDS individuals without HAART or who remain on their first antiretroviral regimen. Of the total, 351 individuals under 40 years and 346 over 40 years were analyzed separately. Subclinical atherosclerosis was assessed by carotid intima-media thickness, using B-mode ultrasound. Multivariate logistic regression was performed to find predictors of subclinical atherosclerosis in the entire group. Subsequent analysis excluded patients with major risk factors for CVD. Magnitudes of associations were expressed by odds ratio (OR) statistical significance, using a 95% confidence interval and *p*-value <0.05.

**Results:**

In the <40 years group subclinical atherosclerosis was associated with male gender (OR: 2.77, 95% CI: 1.43–5.34), nonwhite race (OR: 3.01, 95% CI: 1.23-6.53), obesity (OR: 5.13, 95% CI: 1.79–14.7) and metabolic syndrome (OR: 3.30, 95% CI: 1.44–7.58). In the group ≥40 years predictors of subclinical atherosclerosis were overweight and obesity (OR = 2.53, 95% CI, 0.85–7.54), current CD4 ≥350 cells/mL (OR: 2.81, 95% CI: 1.22–6.47) and NNRTI use ≥ 5 years (OR: 2.65, 95% CI: 1.10-6.37) or PI use >5 years (OR: 1.81, 95% CI: 0.38-8.59). In the multivariate model excluding patients with major risk factors for CVD, age, male sex and nonwhite race were associated with subclinical atherosclerosis in the <40 y group, while in the ≥40 y group, age, HIV viral load >10,000 copies and the use of NNRTI (OR: 7.60, 95% CI: 1.61-35.8) or PI ≥5 years (OR: 3.62, 95% CI: 0.48-26.8) were associated with subclinical atherosclerosis.

**Conclusions:**

In young people the fight against obesity and metabolic syndrome is the main aim in the prevention of CVD. In individuals aged ≥40 y, the prevention of obesity is also of great importance. Moreover, the effects of uncontrolled viremia and the prolonged use of HAART appear to be more harmful in the older group.

## Background

Cardiovascular diseases, especially coronary heart disease (CHD), have been the leading cause of mortality in the general population [1] and have now also been identified as a major cause of death in people with AIDS [2].

However, while the association of atherosclerosis and CHD in itself involves a complex inflammatory process, the acquisition of chronic infection with human immunodeficiency virus (HIV) in the same individual adds further complexity to this process. Thus, we have, firstly, atherosclerosis that begins in childhood, characterized by the presence of lymphocytes, foamy macrophages, increased expression of adhesion molecules, proliferation of smooth muscle cells, fat accumulation in the intima layer of the arteries and plaque maturation lipids in the blood vessels [3], and, secondly, endothelial injury caused by HIV [4] itself and by the action of HAART linked to inflammation [5].

Some authors also mention the great frequency with which the well-known risk factors for CHD, namely a family history of premature atherosclerotic disease, smoking, hypertension, obesity and inactivity [6], are found in these patients.

In addition, metabolic disorders like insulin resistance and dyslipidemia induced by antiretroviral agents such as protease inhibitors and nucleoside analogue reverse transcriptase inhibitors are reported to be predictors of cardiac events in these individuals [7]. Although factors potentially contributing to this increased risk include traditional CHD risk factors and antiretroviral medications, more recent data support a role for inflammatory and immunologic factors as central to a complex mechanism [8]. Decreasing CHD risk among HIV-infected patients is likely to involve the modification of inflammatory and immunologic factors through antiretroviral therapy or other novel strategies, as well as the targeted treatment of traditional CHD risk factors [8-10].

In Brazil, the AIDS epidemic affects young people, predominantly those aged from 20 to 39 years, systematically accounting for over 60% of all reported cases in both sexes [11]. About 250,000 individuals infected with HIV are on combined antiretroviral therapy [12], provided free of charge by the government. However, both the magnitude with which atherosclerosis affects these patients and the impact of the risk factors on the condition remain unknown.

In view of this, the present study, using carotid intima-media thickness (IMT) as a marker of early atherosclerosis in the vascular bed and as a predictor of the development of atherosclerosis [13], was conducted with the following two objectives: firstly, to estimate the frequency with which subclinical atherosclerosis is present in young adults with HIV/AIDS and in those aged 40 years and over; and secondly, to evaluate the association of the traditional risk factors for cardiovascular disease, as well as those related to HIV infection and antiretroviral therapy, with subclinical atherosclerosis in the two groups.

## Methods

### Study population, recruitment, and survey methods

A case–control study was carried out in a population of 697 patients, selected from a cohort of patients with HIV/AIDS being screened for cardiovascular diseases in the state of Pernambuco from July 2007 to January 2010, who were treated at Hospital Oswaldo Cruz (University of Pernambuco) and Hospital Correia Picanço (Pernambuco State Department of Health).

The following inclusion criteria were adopted: age 18 years or older, individuals with no antiretroviral therapy or on their first HAART regimen, absence of opportunistic disease in the three months up to the ultrasound examination and no prior angina, heart failure or myocardial infarction.

*Cases* were defined as individuals over 18 years with subclinical atherosclerosis (IMT greater than 0.8 mm and/or the presence of atheromatous plaques in the carotid arteries identified by ultrasonography). C*ontrols* were those aged over 18 years without subclinical atherosclerosis.

We measured the frequencies of subclinical atherosclerosis and sex, race, schooling, stable partner, traditional risk factors for CHD (smoking, family history of early CHD, arterial hypertension defined according to VIIJC [14], hyperglycemia (fasting glicemia ≥ 100 mg/dL), metabolic syndrome, hypercholesterolemia (≥ 240 mg/dL), low HDL-C (< 40 mg/dL in men and < 45 mg/dL in women) and increased LDL-C (≥ 130 mg/dL), lipodystrophy, hypertriglyceridemia (≥ 150 mg/dL), alcohol use, drug use, as well as the frequency of factors related to HIV/AIDS/HAART (time elapsed since diagnosis of the infection/HIV disease, history of AIDS, viral load counts - at the start of the study, CD4 count <350 cels/mm^3^- nadir and at the start of the study, use and duration of use of the antiretroviral regimen).

The study population was divided into two large groups: those under 40 years of age (<40 y) and those aged 40 years and over (≥40 y), each group being analyzed separately. The reasons for the cut-off point of 40 y were the following: 1) an exploratory analysis of data showed that the chance of atherosclerosis was more than 10 times higher in the 40–49 y age group than in the younger group, and that this chance was even higher in people aged 50 y or over. We thought that, although we could have adjusted our analysis by age, it would be more informative to split the sample into these two groups that might have important differences between them and 2) the 40 y cut-off divided the population of the study down the middle (median age of the population was 40 y).

### Traditional and HIV-related risk factors

To obtain information on risk factors, a questionnaire was applied in individual interviews by trained professionals. We also performed a detailed review of medical records. Measurements of weight, height and waist circumference, as well as blood pressure, were obtained during the interview using the health services’ own equipment.

Serum glucose, total cholesterol, high density lipoprotein (HDL), low density lipoprotein (LDL) and triglycerides assays were performed after blood collection following a fasting period of 12 hours. For triglyceride levels below 400 mg/dL, LDL-cholesterol was calculated using the Freidewald formula: *LDL* = *TC* − [*HDL* + (*TG*/5)] [15].

The CD4 lymphocyte count was performed by flow cytometry (FACSCalibur - Multitest, San Jose, CA) and the viral load determined by the ultrasensitive test (Amplicor HIV-1 Monitor Assay, Roche Molecular Systems, Branchburg, NJ) , whose limits of detection are 50 to 750,000 RNA copies/mL up to six months from the carotid artery ultrasound. The U.S. National Cholesterol Educational Program (NCEP) ATP III [16] definition was used for diagnosis of the metabolic syndrome.

The median time between the USG and the questionnaire was 35 days; between the USG and CD4 and viral load (evaluated simultaneously) it was 96 days; and between the USG and triglycerides /cholestherol (evaluated simultaneously) it was 28 days.

### Measurement of IMT and carotid plaque

The measurements of IMT and determination of atheroma in the extracranial carotid wall were performed using the high-resolution B mode ultrasound equipment GE VIVID Five (Horten, Norway) with a linear transducer (7.5 MHz) and software specifically designed for studying the arteries. The presence of atheroma was defined as a focal prominence greater than 50% of the vessel wall thickness on the adjacent segments and invading the lumen [17]. Measurements of IMT were obtained on the anterior and posterior walls of the right and left carotid [18] arteries as follows: three measurements on the carotid artery one centimeter proximal to the bifurcation, one measurement in the carotid bulb and two measurements in the first inch of the internal carotid artery. For the final analysis we used the maximum mean of the thickness of each segment. Subclinical atherosclerosis was considered to be present when the thickness of the carotid intima-media was ≥ 0.8 mm and/or in the presence of an atheroma plaque in any carotid segment. We chose the same cut-off of 0.8 mm for evaluation of carotid atherosclerosis as that used by Jerico et al. [19]. This choice was based on the fact that the measurement of the common carotid IMT in a normal population of young adults produces values lower than 0.8 mm [20], with an increase from 0.01 to 0.02 mm per year. In addition, individuals with a carotid IMT lower than 0.8 mm have less risk of cardiovascular and cerebrovascular events (<4.6%) in six years [21]. Given that the median age of the study population was 40 years, the 0.8 mm cut-off seemed to be a rational choice.

To minimize bias, the following measures were adopted: a precodified standardized questionnaire and training of all the interviewers in order to standardize techniques; selection of controls from the same population established for cases, provided that both groups satisfied the same inclusion and exclusion criteria; and masking the ultrasound technicians in relation to the traditional risk factors for cardiovascular disease (CVD), status and HIV therapy.

For quality control of the imaging examination 480 repeated measurements of IMT thickness were made, resulting in an inter-class correlation coefficient of 0.866 and a Kappa concordance index of 0.895.

### Data analysis

Univariate and multivariable logistic regression analysis was performed using the statistical package STATA version 9.0 to identify potential predictors of subclinical atherosclerosis, assessed separately in the two groups (<40 y and 40≥ y).

The insertion of the data was carried out by double entry, under weekly supervision, thus avoiding transcription errors. Backup copies of the definitive database were made on CD-ROM and subsequently submitted to consistency tests.

Medians were compared using the nonparametric test of Kruskall-Wallis. Comparisons between the groups of categorical variables were performed using the chi-square test and, when necessary, the Fisher Exact test. Logistic regression was used to calculate crude and adjusted odds ratios of the association between the independent variables and the outcome, with the respective 95% confidence intervals (CI) and p-values (likelihood ratio statistic).

The data analysis was carried out in three steps: 1) univariate analysis of each group to determine the variables closely associated with the outcome: variables associated with the outcome with P < 0.20 in the univariate analysis were successively included in a multivariate logistic regression model; 2) final multivariate model: the variables selected in the previous step were inserted into the final multivariate model using the forward selection (including the entire group of patients), and those with a *p*-value ≤ 0.10 remained in the final model; and 3) a subsequent multivariate logistic regression of factors associated with subclinical atherosclerosis was carried out with the exclusion of patients with traditional metabolic risk factors for CVD and smoking, using a forward strategy similar to that used previously.

Several models were tested in the multivariable analysis, and in those which included the metabolic syndrome variable (composite variable) the variables comprising this syndrome were not included in the analysis.

The study was approved by the Ethics and Research Committee of the University of Pernambuco (UPE) and free and informed written consent was obtained from all participants.

## Results

### Characteristics of the study participants according to age group

A total of 697 individuals were studied, 351 in the < 40 y group and 346 in the ≥ 40 y group (Table 1). The mean age was 31.8 years in the former and 47.8 years in the latter. There were no differences between groups regarding sex distribution, proportion of white race, smoking or drug use. A total of 312 individuals had subclinical atherosclerosis, of whom 82 (23.4%) were in the < 40 y group, whose mean IMT thickness was 0.742 mm, and 230 (66.5%) in the ≥ 40 y group, whose mean IMT thickness was 0.841 mm, representing a statistically significant difference between the groups (p <0.001).

**Table 1:** Comparison of qualitative and quantitative variables in HIV-infected individuals according to age group, followed in two referral hospitals in the state of Pernambuco, Brazil

**Variables**	**< 40 years (n=351)**	**≥ 40 years (n=346)**	***p-value***
**Sociodemographics**			
Age (years) – Mean (SD)	31.8 (4.96)	47.8 (6.14)	< 0.001
Male (%)	59.5	57.8	0.641
White (%)	25.6	24.9	0.811
Stable partner (%)	19.4	24.3	0.117
**Habits**			
Smoking (%)	23.0	22.0	0.725
Alcohol consumption (%)	40.4	35.2	0.167
Injecting drugs use (%)	6.0	3.8	0.368
**Clinical characteristics**			
Diabetes (%)	0.9	6.2	< 0.001
Lipodystrophy (%)	31.5	39.8	0.024
Dyslipidemia (%)	16.1	31.7	< 0.001
Metabolic syndrome (%)	16.9	31.3	< 0.001
**HIV-related characteristics**			
Time elapsed since diagnosis HIV (in months -DP)	40.5 (37.5)	58.4 (49.6)	< 0.001
CD4 T-lymphocytes baseline - Median (P_25_ – P_75_)	323 (158 – 498)	258 (94 – 473)	0.005*
Viral load baseline - Median (P_25_ – P_75_)	29600 (3341 – 134000)	57400 (6170 – 205000)	0.026*
CD4 T-lymphocytes current - Median (P_25_ – P_75_)	391 (262 – 535)	383 (242 – 564)	0.949*
Viral load, current undetectable (%)**	31.7	46.7	0.005
HAART duration (in months) Median (P_25_–P_75_)	23.5 (9.9 – 42.6)	36.3 (17.7 – 65.2)	< 0.001*
NNTRI Use (%)	36.6	47.1	0.005
PI use (%)	21.7	26.9	0.112
Without HAART (%)	41.3	25.7	< 0.001
**Laboratory tests**			
Total cholesterol (mg/dL) - Mean (SD)	163.5 (40.0)	189.6 (54.5)	< 0.001
LDL cholesterol (mg/dL) - Mean (SD)	93.9 (38.8)	106.7 (40.8)	0.001
HDL cholesterol (mg/dL) **-** Mean (SD)	42.2 (13.6)	43.5 (14.1)	0.216
Triglycerides (mg/dL) - Mean (SD)	148.8 (102.8)	204.6 (213.0)	< 0.001
**USG of carotid arteries**			
Presence of sublinical atherosclerosis (%)	23.4	66.5	< 0.001
Carotid intima thickness (mm) - Median (SD)	0.742 (0.084)	0.841 (0.150)	< 0.001

* Kruskal - Wallis test.

** 328 patients with viral load information.

Diabetes (6.2%), lipodystrophy (39.8%), metabolic syndrome (31.3%) and dyslipidemia (31.7%) were more frequent in the ≥ 40 y group. The mean values of total cholesterol (189.6 mg/dL), LDL cholesterol (106.7 mg/dL) and triglycerides (204.6 mg/dL) were higher in the ≥ 40 y group, except for mean HDL cholesterol, for which there were no statistical significant differences between the groups (p = 0.216).

A total of 31.7% of those on HAART in the < 40 y group had an undetectable viral load, and the corresponding figure was 46.7% in the ≥ 40 y group (p = 0.005), with a median of CD4 391 cells/μl in the former group and 383 cells/μl in the latter. The < 40 y group had been using HAART for less time (median, 23.5 months) than the ≥ 40 y group (median, 36.3 months) (p <0.001).

### Values of carotid IMT in the two different age groups

In the group of patients < 40 years the median of the carotid IMT was higher in patients on HAART than in those without therapy (p=0.069) and in those with duration of use of HAART ≥5 years (p=0.015), while no differences were found in patients in different strata of CD4, viral load and types of antiretroviral regimen. In the ≥ 40 y group carotid IMT was greater in patients with higher CD4 strata (p=0.011), and in those on HAART for ≥ 5 years (0.070). There were no differences in the carotid IMT in all viral load strata and different types of antiretroviral regimen (Table 2).

**Table 2:** Values of the carotid intima media thickness (IMT) of the patients < 40 years and ≥ 40 years

**Variables**	**< 40 y group**		**≥ 40 y group**	
	**Carotid MIT** Median (P_25_; P_75_)	***p-value***	**Carotid MIT** Median (P_25_; P_75_)	***p-value***
HAART use		**p = 0.069**		p = 0.166
Yes	0.735 (0.691; 0.792)		0.840 (0.772; 0.917)	
No	0,722 (0.677; 0.780)		0.827 (0.740; 0.915)	
CD4 T-lymphocytes (cells/mm^3^)		p = 0.373		**p = 0.011**
<200	0.720 (0.685; 0.782)		0.797 (0.742; 0.964)	
200 to 350	0.728 (0.681; 0.792)		0.822 (0.760; 0.919)	
≥ 350	0.731 (0.693; 0.779)		0.852 (0.802; 0.920)	
Viral load (copies/mL)		p = 0.269		p = 0.616
< 50	0.745 (0.677; 0.822)		0.836 (0.765; 0.135)	
50 to < 10.000	0.742 (0.705; 0.795)		0.837 (0.757; 0.940)	
10.000 to < 100.000	0.723 (0.681; 0.769)		0.852 (0.763; 0.922)	
≥ 100,000	0.690 (0.682; 0.741)		0.831 (0.775; 0.918)	
Types HAART regimens		p = 0.109		p = 0.132
No HAART use	0.722 (0.677; 0.780)		0.827 (0.740; 0.915)	
PI	0.733 (0.690; 0.767)		0.782 (0.732; 0.875)	
NNRTI	0.729 (0.696; 0.784)		0.837 (0.778; 0.897)	
Duration of use of HAART		**p = 0,015**		**p = 0.070**
No use	0.722 (0.677; 0.780)		0.827 (0.740; 0.915)	
< 5 years	0.730 (0.690; 0.790)		0.830 (0.757; 0.918)	
≥5 years	0.757 (0.730; 0.815)		0.864 (0.805; 0.915)	

### Association between subclinical atherosclerosis and demographic, clinical, laboratory and HIV-related characteristics in the different age groups

Tables 3 and 4 summarize the results of the univariate analysis in relation to the association of the variables with the presence of subclinical atherosclerosis. In the < 40 y age group, male gender, nonwhite race, stable partner, hypertension, cholesterol ≥ 240 mg/dL, triglicerydes ≥ 150 mg/dL, fasting glucose ≥ 100 mg/dL, waist circumference, overweight and obesity were significantly associated with subclinical atherosclerosis after adjustment for age. No significant differences were found with other risk factors for CVD such as smoking and HDL or LDL-cholesterol. The ≥ 40 y group presented a statistically significant difference for age, stable partner, hypertension, overweight, obesity and, and also for altered levels of total cholesterol and LDL cholesterol. As regards the variables related to HIV, a statistically significant association was observed with CD4 counts > 350 cells/mL, the use of nonnucleoside reverse-transcriptase inhibitors (NNRTIs) for more than 5 years. However we found no significant differences in the virological status of HIV.

**Table 3:** Univariate analysis of the association between subclinical atherosclerosis and socio-demographic variables, habits, and metabolic data in individuals infected with HIV by age group, seen at two referral hospitals in the state of Pernambuco, Brazil

**Variables**	**< 40 years old (n=351)**			**≥ 40 years old (n=346)**		
	**% subclinical atherosclerosis**	**OR(CI)**	***p*-value**	**% subclinical atherosclerosis**	**OR(CI)**	***p*-value**
**Age (continuous)**	-	**1.17 (1.10 – 1.25)**	**< 0.001**	-	**1.11 (1.06 – 1.16)**	**< 0.001**
**Gender**						
Female	18.3	1.0	-	62,7	1.0	**-**
Male	26.8	**1.63 (0.96 – 2.75)**	**0.067**	69.2	**1.33 (0.84 – 2.09)**	**0.213**
**Race**						
White	13.3	1.0	-	68.2	1.0	-
Nonwhite	26.8	**2.38 (1.22 – 4.64)**	**0.011**	65.9	**0.89 (0.53 – 1.52)**	**0.691**
**Schooling (years of study)**						
1 to 12	22.3	1.0	-	66.4	1.0	-
13 or more	32.3	**1.67 (0.77 – 3.59)**	**0.190**	68.3	**1.10 (0.58 – 2.10)**	**0.759**
**Stable partner**						
No	20.1	1.0	-	62.5	1.0	-
Yes	36.8	**2.30 (1.30 – 4.08)**	**0.004**	78.6	**2.19 (1.23 – 3.91)**	**0.008**
**Smoking**						
No	24.4	1.0	-	66.4	1.0	-
Yes	19.8	**0.87 (0.36 – 2.09)**	**0.759**	66.7	**0.97 (0.46 – 1.93)**	**0.929**
**Lipodystrophy**						
No	20.6	1.0	-	63.5	1.0	-
Yes	28.0	**1.50 (0.88 – 2.54)**	**0.131**	70.2	**1.35 (0.84 – 2.17)**	**0.206**
**Metabolic Syndrome**						
No	17.9	1.0	-	63.9	1.0	-
Yes	49.1	**4.42 (2.44 – 7.99)**	**0,000**	72.0	**1.44 (0.87 – 2.38)**	**0.147**
**Hypertension**						
No	19.1	1.0	**-**	61.0	1.0	-
Yes	44.3	**3.36 (1.87 – 6.03)**	**0.000**	76.9	**2.12 (1.28 – 3.52)**	**0.004**
**Waist circumference (cm)**						
Normal	19.2	1.0	-	62.3	1.0	-
Abnormal*	41.2	**2.94 (1.66 – 5.18)**	**0.000**	74.7	**1.64 (0.93 – 2.89)**	**0.087**
**BMI (kg/m**^**2**^**)**						
< 25	15.5	1.0	**-**	60.0	1.0	-
≥ 25 and < 30	32.6	**2.63 (1.49 – 4.67)**	**0.001**	75.2	**2.02 (1.19 – 3.42)**	**0.008**
≥ 30	52.9	**6.14 (2.86 – 13.2)**	**0.000**	81.2	**2.88 (1.13 – 7.32)**	**0.025**
**Total cholesterol (mg/dL)**						
< 200	21.7	1.0	-	62.6	1.0	-
200 - <240	28.6	**1.44 (0.69 – 2.98)**	**0.323**	69.4	**1.35 (0.79 – 2.32)**	**0.269**
≥ 240	42.1	**2.62 (1.01 – 6.80)**	**0.048**	83.0	**2.89 (1.22 – 6.84)**	**0.015**
HDL-cholesterol (mg/dL) **						
Normal	24.8	1.0	-	64.8	1.0	-
Low	22.4	**0.87 (0.52 – 1.44)**	**0.596**	67.7	**1.13 (0.72 – 1.79)**	**0.579**
**LDL-cholesterol (mg/dL)**						
< 130	22.2	1.0	-	61.5	1.0	-
≥ 130	30.4	**1.53 (0.77 – 3.05)**	**0.225**	82.9	**3.02 (1.58 – 5.82)**	**0.001**
**Triglycerides (mg/dL)**						
< 150	18.9	1.0	-	63.9	1.0	-
≥ 150	31.4	**1.96 (1.17 - 3.28)**	**0.010**	69.3	**1.27 (0.80 – 2.01)**	**0.296**
**Fasting glucose (mg/dL)**						
< 100	21.6	1.0	-	65.6	1.0	-
≥ 100	35.7	**2.01 (1.01 – 4.01)**	**0.046**	72.4	**1.39 (0.81 – 2.38)**	**0.227**

*Men: waist >102 cm; women: waist >88 cm.

**Low HDL-cholesterol < 40 mg/dL in men and < 45 mg/dL in women.

**Table 4:** Univariate analysis of the association between subclinical atherosclerosis and other factors related to HIV, by age group, seen at two referral hospitals in the state of Pernambuco, Brazil

**Variables**	**< 40 years old (n=351)**			**≥ 40 years old (n=346)**		
	**% subclinical atherosclerosis**	**OR(CI)**	***p*-value**	**% subclinical atherosclerosis**	**OR(CI)**	***p*-value**
**Time elapsed since HIV diagnosis (years)**						
< 1	19.3	1.0	-	55.1	1.0	-
1 to < 5	22.3	**1.19 (0.64 – 2.25)**	**0.570**	65.8	**1.56 (0.81 – 3.01)**	**0.179**
≥5	30.5	**1.82 (0.91 – 3.66)**	**0.090**	70.6	**1.95 (0.99 – 3.83)**	**0.051**
**Clinical history of AIDS**						
No	24.0	1.0	-	65.7	1.0	-
Yes	24.2	**1.01 (0.58 – 1.76)**	**0.975**	72.7	**1.38 (0.68 – 2.81)**	**0.362**
**CD4+ T-lymphocytes (cells/mm**^**3**^**)**						
< 200	22.2	1.0	-	53.3	1,0	-
200 to < 350	24.4	**1.12 (0.51 – 2.48)**	**0.763**	63.1	**1.49 (0.81 – 2.74)**	**0.193**
350 and more	21.9	**0.98 (0.42 – 2.30)**	**0.963**	84.0	**4.59 (2.19 – 9.59)**	**0.000**
**Viral load (copies/mL)**						
< 50	29.4	1.0	**-**	67.5	1.0	**-**
50 to < 10,000	24.6	**0.78 (0.34 – 1.81)**	**0.566**	62.7	**0.89 (0.41-1.91)**	**0.767**
10,000 < 100,000	19.4	**0.58 (0.21 – 1.61)**	**0.295**	72.7	**1.61 (0.54-4.73)**	**0.384**
> 100,000	15.4	**0.44 (0.09 – 2.21)**	**0.316**	68.6	**1.07 (0.32-3.55)**	**0.905**
**HAART use**						
No	19.9	1.0	**-**	59.5	1.0	**-**
Yes	26.0	**1.42 (0.85 – 2.36)**	**0.184**	68.9	**1.50 (0.91 – 2.48)**	**0.109**
**Types HAART regimens**						
No HAART use	19.9	1.0	**-**	59.5	1.0	**-**
PI	17.1	**0.83 (0.40 – 1.71)**	**0.619**	59.3	**0.99 (0.54 – 1.79)**	**0.977**
NNRTI	31.2	**1.83 (1.05 – 3.19)**	**0.031**	74.2	**1.95 (1.12 – 3.39)**	**0.017**
**HAART duration of use (years)**						
No HAART use	19.9	1.0	**-**	59.5	1.0	**-**
< 5	24.7	**1.32 (0.78 - 2.25)**	**0.299**	64.2	**1.21 (0.72 – 2.06)**	**0.460**
≥ 5	34.6	**2.13 (0.86 – 5.27)**	**0.100**	81.6	**3.00 (1.46 – 6.16)**	**0.003**

The multivariate model of the associations of demographic factors, traditional risk factors for CVD and HIV infection with the presence of subclinical atherosclerosis in 697 patients is presented in Table 5. The independent predictors of subclinical atherosclerosis in the < 40 y group were age, male sex, nonwhite race, stable partner, obesity and metabolic syndrome, and in the ≥ 40 y group they were age, male sex, stable partner, obesity, total cholesterol > 240 mg/dL, CD4 lymphocyte count > 350 cells/μl and duration on NNRTI ≥ 5 years. Obesity and hypertension presented an association with the presence of subclinical atherosclerosis, but nullified each other when introduced into the multivariate model. Thus hypertension was withdrawn from the final model, since obesity is related to the genesis of hypertension.

**Table 5:** Multivariate model of the association between subclinical atherosclerosis with sociodemographic variables, habits, family history, clinical parameters, lipid profile, fasting glucose and features related to HIV by age group including all patients (total of 694 patients)

**Variables**	**< 40 years old**		**≥ 40 years old**	
	**OR(IC)**	***p*-value**	**OR(CI)**	***p-*value**
**Age (continuous)**	1.12 (1.05 – 1.20)	**0.001**	1.11 (1.05 – 1.17)	**< 0.001**
**Male sex**	2.77 (1.43 – 5.34)	**0.002**	1.62 (0.94 – 2.81)	**0.080**
**Nonwhite**	3.01 (1.23 - 6.53)	**0.007**	1.37 (0.73 – 2.57)	0.321
**BMI overweight**	1.65 (0.79 – 3.44)	0.344	1.93 (1.04 – 3.57)	**0.036**
Obese	5.13 (1.79 – 14.7)	**0.002**	2.53 (0.85 – 7.54)	**0.095**
**Metabolic Syndrome**	3.30 (1.44 – 7.58)	**0.005**	1.01 (0.54 – 1.88)	0.968
**Total cholesterol <200**	1.0		1.0	
200 to < 240 mg/dL	0.83 (0.32 - 2.03)	0.701	1.24 (0.66 – 2.32)	0.491
≥ 240 mg/dL	2.28 (0.83 – 9.40)	**0.095**	2.14 (0.76 – 5.98)	**0.145**
**Stable partner**	2.07 (1.03 – 4.19)	**0.041**	2.41 (1.24 – 4.67)	**0.009**
**CD4+ T-lymphocytes <200**	1.0		1.0	
200 to < 350 cels/mm^3^	0.86 (0.33 – 2.24)	0.758	1.18 (0.59 – 2.37)	0.631
≥ 350 cels/mm^3^	0.71 (0.24 – 2.09)	0.542	2.81 (1.22 – 6.47)	**0.015**
**HAART duration of use**				
No HAART use	1.0		1.0	
PI < 5 years	0.64 (0.27 – 1.54)	0.323	0.67 (0.32 – 1.39)	0.287
PI ≥ 5 years	2.15 (0.23 – 20.1)	0.503	1.81 (0.38 – 8.59)	0.453
NNRTI < 5 years	1.52 (0.74 – 3.08)	0.248	1.31 (0.66 – 2.60)	0.426
NNRTI ≥ 5 years	1.94 (0.62 – 6.07)	0.254	2.65 (1.10 – 6.37)	**0.028**

In order to verify if an HIV-related effect was masked by traditional risk factors for atherosclerosis two new multivariate models were prepared. The first model was prepared excluding the individuals with the major traditional risk factors that were significant in the overall final model, entitled **Model A**, resulting in the exclusion of 333 patients as follows: patients with metabolic syndrome (n=166); obese individuals (n=20); hypertensives (n=77); patients with total cholesterol ≥ 240 mg/dL (n=24) and smokers (n=46). The results of the multivariate **Model A** demonstrated that, in the <40 y group, age, male sex and nonwhite race were associated with subclinical atherosclerosis. In the ≥ 40 y group, age, viral load ≥ 10,000 or 100,000 copies RNA/mL and time on PI ≥ 5 years (OR: 3.62) or time on NNRTI ≥ 5 years (OR: 7.60) remained as factors independently associated with carotid atherosclerosis. The other multivariate model, entitled **Model B**, comprised all 333 patients with the major traditional risk factors for cardiovascular disease excluded from Model A. In this multivariate final Model B, among patients ≥ 40 years, those with CD4 levels ≥ 350 lymphocytes/mm^3^ had a 10.8 higher risk (95% CI: 2.89 – 40.4; p < 0.001) of presenting subclinical atherosclerosis. Viral load was not associated with atherosclerosis in this group. The use of HAART was not related to atherosclerosis, except for the use of NNRTI < 5 years.

## Discussion

In our study differences in the magnitude and significance of associations between risk factors and subclinical atherosclerosis were observed between the groups, starting with the higher frequency of atherosclerosis (66.5%) in the ≥ 40 y group than in the < 40 y group (44.8%). In patients with HIV/AIDS in the < 40 y group it was the traditional risk factors such as male sex, nonwhite race, obesity, metabolic syndrome and hypercholesterolemia that were associated with subclinical atherosclerosis. In the ≥ 40 y group, male sex, overweight and obesity were also associated with subclinical atherosclerosis; however, factors related to HIV and HAART (CD4 above 350 cells/mL and use of NNRTI for at least 5 years) were also associated with subclinical atherosclerosis in this age group. Age and stable partner were independent predictors of subclinical atherosclerosis in both groups. Analyzing only patients without major modifiable risk factors related to cardiovascular disease, only age, male sex and nonwhite race seemed to be important with regard to the presence of subclinical atherosclerosis in the < 40 y group, while in the ≥ 40 y group, age, viral load and the use of NNRTI ≥ 5 years were independently associated with the presence of atherosclerosis. In the group of persons with at least one modifiable risk factor for CVD and ≥ 40 y, patients with levels ≥ 350 lymphocytes/mm3 had a 10.8 higher risk of having subclinical atherosclerosis.

Understanding the risk of cardiovascular disease in individuals with HIV is a complex matter. Since the introduction of HAART, cardiovascular events resulting from atheromatous complications have been reported [22,23], but it is not known to what extent this risk is attributable to the therapy and its adverse effects or to genetic factors, traditional risk factors or even to the inflammatory state associated with HIV [24].

The measurement of CMIT thickness has been increasingly used as a surrogate marker of atherosclerosis due to the long time required for the appearance of clinical events. A systematic review and meta-analysis showed the high predictive capacity of the measurement of CMIT to predict future cardiovascular events such as myocardial infarction and stroke in the general population [25]. The CMIT thickness among HIV-positive patients is higher than among HIV-negative ones and HIV infection is an independent risk factor for the presence of carotid thickening [26-28] and its progression [26].

Our study aimed to examine whether the risk factors associated with increased thickening of CMIT in HIV-infected individuals are different between individuals aged below 40 years and those 40 years or more. This question arose as the exploratory analysis of our data showed that the chance of atherosclerosis was more than 10 times higher in the 40–49 y age group than in the younger group, and that this chance was even higher in people aged 50 y or over. We decided that, although we could have adjusted our analysis by age, it would be more informative to split the sample into these two groups, bearing in mind that there might be important differences between them. Another point reinforced our decision, namely the fact that the 40 y cut-off divided our population down the middle (the median age of the population was 40 y).

Our overall results showed that both traditional and new risk factors (albeit not related to HIV) were strongly associated with the onset of carotid thickening, particularly in the younger population. Thus, male sex, non-white race, obesity, metabolic syndrome and elevated levels of total cholesterol were associated with the presence of thickening of CMIT in the group < 40 y. This result is in agreement with studies that assessed thickening of CMIT in HIV-positive individuals, which also found the traditional risk factors related to CVD to be the main factors associated with increased CMIT [24,26,29].

The metabolic syndrome was detected in 16% of the younger group and in 31% of ≥ 40 y group of our study. In the general population, the metabolic syndrome is associated with a 2.35-fold greater risk of developing CVD [30] and was an independent factor associated with greater CIMT in younger patients in our study. In the population with HIV, the metabolic syndrome is described as a possible complication of the use of HAART, especially in regimens containing a protease inhibitor [31], and might or might not be suggestive of an indirect effect of HAART in atherogenesis. Nonetheless, in an analysis that included 331 antiretroviral treatment-naive patients conducted by Stein et al., the metabolic syndrome was also an important factor in the presence of carotid lesions [32], suggesting that the role of the metabolic syndrome in CMIT thickening is not necessarily related to the use of HAART.

With regard to obesity as a predictor of CVD risk, our study was in agreement with the literature in an at-risk population not infected by HIV, which reported a linear relationship with thickening of the CIMT in young obese individuals [33].

In the final model of the multivariable analysis of all patients in each age group, no factor related to HIV or its treatment was associated with CMIT thickening in the < 40 y group. On the other hand, the group of individuals 40 years or more, levels of CD4 > 350 lymphocytes/mm3 and the use of NNRTI > 5 years were associated with CMIT thickening.

One hypothesis is that the role of the traditional risk factors is so important that it was not possible to identify factors specifically related to HIV or to HAART as being associated with the disease, particularly in the younger group. This question was assessed by excluding all the patients that presented at least one modifiable risk factor unrelated to HIV (obesity, metabolic syndrome, hypertension, cholesterol above 240mg/dL and smoking) in order to check whether some factor related to HIV could be masked. In this important analysis (Table 6, Model A) we found that among patients < 40 y the results were similar, with no association between CMIT thickening and HIV-related factors.

**Table 6:** Multivariate model of factors related to the presence of subclinical atherosclerosis among 361 individuals without the main risk factors for cardiovascular disease (CVD) (excluding a total of 333 patients = 166 patients with metabolic syndrome, 20 obese patients, 77 hypertensive patients, 24 individuals with total cholesterol ≥ 240 mg /dL and 46 smokers) (Model A) and among 333 patients with risk factors for cardiovascular diseases (Model B)

**Variables**	**Multivariate Model A (n=361)**				**Multivariate Model B (n=333)**			
	**< 40 years old (n=218)**		**≥ 40 years old (n=143)**		**< 40 years old (n=133)**		**≥ 40 years old (n=200)**	
	**OR(CI)**	***p*-value**	**OR(CI)**	***p-*value**	**OR(CI)**	***p*-value**	**OR(CI)**	***p-*value**
**Age (continuous)**	1.09 (1.00 – 1.19)	**0.037**	1.15 (1.06 – 1.25)	**0.000**	1.21 (1.08 – 1.36)	**0.001**	1.12 (1.04 – 1.21)	**0.003**
**Male sex**	3.02 (1.20 – 7.59)	**0.018**	1.03 (0.45 – 2.34)	0.937	1.59 (0.64 – 3.95)	0.311	**2.53** (1.16 – 5.51)	**0.019**
**Nonwhite race**	3.21 (0.90 – 11.4)	**0.071**	1.23 (0.42 – 3.67)	0.702	**2.59** (0.96 – 6.99)	**0.060**	1.28 (0.55 - 2.99)	0.557
**Stable partner**	1.62 (0.64 – 4.08)	0.306	**3.97** (1.46 – 10.8)	**0.007**	**3.14** (1.13 – 8.72)	**0.028**	2.04 (0.78 – 5.36)	0.144
**CD4+ T-lymphocytes <200**	1.0		1.0		1.0		1.0	
200 to < 350 cels/mm^3^	1.07 (0.35 – 3.23)	0.902	0.97 (0.34 – 2.79)	0.967	0.51 (0.07 – 3.38)	0.486	**2.52** (0.85 – 7.48)	**0.094**
≥ 350 cels/mm^3^	0.49 (0.12 – 1.98)	0.321	2.41 (0.68 – 8.47)	0.168	0.82 (0.11 – 5.79)	0.847	**10.8** (2.89 – 40.4)	**0.000**
**Undetectable Viral Load**	1.0		1.0		1.0		1.0	
50 to < 10,000	0.96 (0.24 – 3.78)	0.954	2.16 (0.50 – 9.37)	0.300	0.48 (0.09 – 2.40)	0.378	0.54 (0.15 – 1.98)	0.355
10,000 to < 100,000	0.47 (0.07 – 2.93)	0.425	**5.95** (1.00 – 35.3)	**0.050**	0.83 (0.11 – 6.28)	0.863	1.46 (0.23 – 9.11)	0.681
≥ 100,000	0.33 (0.02 – 4.08)	0.394	**6.84** (0.90 – 51.8)	**0.063**	0.27 (0.00 –12.77)	0.512	0.91 (0.10 - 7.80)	0.933
**HAART duration of use**								
No HAART use	1.0		1.0		1.0		1.0	
IP < 5 years	0.51 (0.15 – 1.75)	0.290	0.47 (0.137 – 1.66)	0.245	0.75 (0.19 – 2.87)	0.682	**1.72** (0.60 – 4.89)	0.307
IP ≥ 5 years	-	-	**3.62** (0.48 – 26.8)	0.208	0.87 (0.05-15.65)	0.925	-	
NNRTI < 5 years	0.55 (0.18 – 1.62)	0.281	1.79 (0.57 – 5.59)	0.316	**2.84** (0.92-8.68)	**0.067**	**2.01** (0.71 – 5.71)	0.189
NNRTI ≥ 5 years	**2.41** (0.59 – 9.82)	0.218	**7.60** (1.61 – 35.8)	**0.010**	0.24 (0.02-2.53)	0.236	**2.04** (0.63 - 6.58)	0.229

However important HIV-related factors in the group of patients ≥ 40 y became even clearer after the exclusion of patients with modifiable risk factors unrelated to HIV. The first such finding was the association of the higher CIMT with the use of antiretroviral therapy for more than 5 years. This finding was particularly evident when using NNRTI > 5 years (OR: 7.6), but was also suggested when using PI > 5 years (OR: 3.62) although the low number of patients using PI > 5 years in this group did not permit any firm conclusion (only 16 observations).

The second important finding was the association of thickening of CMIT with viremia ≥ 10.000 copies. The levels of association were shown to be higher the higher the category of viral load. HIV viremia is related to inflammation [8,24], a potent trigger for atherosclerosis [3,8]. This finding is in agreement with that obtained by Baker *et al*., who assessed the factors associated with progression of CMIT thickening in 389 HIV-infected patients after adjustment for traditional risk factors. They found that low baseline viral load (< 400 RNA copies / mL) and the maintenance of viral load supression have been associated with lower thickening of CMIT after a period of 2 years [34].

Thus, despite the heavy weight of traditional factors in the presence of carotid thickening, the chronic use of HAART and high levels of viremia are independent factors for its occurrence, particularly in patients aged 40 years or more.

It might be assumed that a vessel in the process of atherosclerosis "inherent in aging” would suffer a greater impact with the prolonged use of HAART than the still “healthy” vessels of the young. It might also be assumed that there is a synergic action of the process of atherosclerosis “inherent in aging”, together with other factors related to the therapy that are yet to be clearly identified. However, the association of atherosclerosis specifically with the use of a PI suggested in previous studies was not observed in our population in either group of patients. This result was consistent with CMIT data from 337 patients, in whom no association was found between the use of PI and CMIT thickening [35], and with the findings of a meta-analysis that evaluated the association of regimens containing PI and subclinical atherosclerosis and, as in our study, revealed no associations with this group of drugs [36].

However two of the results related to the use of HAART in the group aged < 40 y deserve attention: a) there was an association between the median of IMT and HAART use (with a borderline p-value) and duration of use of HAART, though the median value of IMT was below the cut-off point adopted in our study; and b) there was no statistically significant association between HIV-related factors and subclinical atherosclerosis, though the lack of association with use of NNRTI > 5 years may be due to a problem of power, as suggested by the width of the confidence interval. These data suggest that the use and type of HAART may have some impact on the development of atherosclerosis in this age group, an aspect of the present study that does not allow any firm conclusions.

Our study found an association between a high lymphocyte count and a greater chance of presenting subclinical atherosclerosis in the ≥ 40 y group; however, this effect disappeared after we excluded those patients with metabolic risk factors for CVD. One possible explanation is that patients who were immunologically compensated (particularly when using HAART), i.e. with high levels of CD4 lymphocytes, are more liable to have traditional metabolic complications such as obesity, metabolic syndrome and hypertension and, consequently, a higher risk of presenting subclinical atherosclerosis [37,38]. Hsue has shown that the rate of progression of CMIT thickening was predicted by the CD4 nadir count [26]. However, as in our findings, baseline, nadir or present CD4 lymphocytes count were not associated with the progression of CMIT thickness in the study by Baker (2011) [34] or with CMIT thickness in antiretroviral naïve individuals in the study by Stein (2012) [24].

Finally, in the older group, obesity was also a major factor associated with atherosclerosis, as in the younger one, which should alert us to the importance of fighting it in HIV patients of all ages.

All the studies reported in the literature, including our own, have shown that age is an independent factor associated with CMIT thickening [24,34,35]. Nonetheless, unlike previous studies, in which the authors adjusted the risk factors for CMIT by age in their final model of multivariable analysis [24,34], the population in our study was split into two groups, an approach that clearly shows the differences in their respective risk factors.

This study has the limitations of not having included controls not infected with HIV and of not having assessed the intensity of immune activation using biomarkers of inflammation. Another limitation related to the nature of the study is that time is an important factor that was not addressed. Those < 40 y probably had less exposure to both traditional and HIV-related risk factors. For example, taking a hypertensive participant from both groups and assuming the hypertension (or HIV for that matter) developed at about the same age, the participant in the > 40 y group would have had longer exposure to hypertension (or viremia). This is important in that it distinguishes between hypertension (or viremia) as a relevant risk factor in the < 40 y group as opposed to exposure to hypertension over time as a risk factor. Given the nature of the study, it is unlikely that this effect can be accounted for statistically.

However one major advantage of our study compared to others should be emphasized, namely the inclusion of individuals on a single antiretroviral regimen, that is, with no change in the class of drug throughout the treatment, a feature that ensures the "purity" of the study with regard to the evaluation of the association of drug classes with atherosclerosis. Another advantage was the elaboration of a model excluding patients with major risk factors for atherosclerosis, for this offers an opportunity to specifically examine the immunologic, virologic and therapeutic factors involved in subclinical atherosclerosis.

In conclusion, the findings of our study suggest that the preventive approach should have somewhat different goals in the prevention of CVD in patients with HIV/AIDS in different age groups. In young people, especially males, the fight against obesity, hypercholesterolemia and metabolic syndrome are the main aims of prevention. In individuals aged 40 years and over, the prevention of obesity and hypercholesterolemia is also of great importance. Moreover, the effects of uncontrolled viremia and the prolonged use of HAART appear to be more harmful in the older group. New therapeutic strategies with no atherogenic effects are urgently needed.

## Competing interests

The authors declare that they have no competing interests.

## Authors' contributions

VMGA carried out the carotid ultrasounds, conceived the study and drafted the manuscript. JCZ and DBMF recruited, selected and followed the patients. MFPMA prepared the questionnaire. RAAX participated in the design, coordinated and supervised the statistical analysis. URM performed the statistical analysis. HRL conceived the study, participated in the design, coordinated and drafted the manuscript. All authors read and approved the final manuscript.

## Pre-publication history

The pre-publication history for this paper can be accessed here:

http://www.biomedcentral.com/1471-2334/13/274/prepub
